# Establishment of an Infrared-Camera-Based Home-Cage Tracking System Goblotrop

**DOI:** 10.1523/ENEURO.0218-25.2025

**Published:** 2025-10-23

**Authors:** Theo Gabloffsky, Katharina Schuster, Annelie Zimmermann, Anna Staffeld, Alexander Hawlitschka, Ralf Salomon, Linda Frintrop

**Affiliations:** ^1^Institute of Applied Microelectronics and Computer Engineering, University of Rostock, Rostock 18051, Germany; ^2^Institute of Anatomy, Rostock University Medical Center, Rostock 18057, Germany

**Keywords:** animal behavior, anorexia nervosa, artificial intelligence, circadian rhythm, locomotor activity

## Abstract

Studying locomotor activity in animal models is crucial for understanding physiological, behavioral, and pathological processes. This study aimed to develop an artificial intelligence-based tracking system called Goblotrop, designed to localize rodents within their laboratory environment. The Goblotrop system uses two infrared cameras to record videos of rodents in their home cages. A neural network analyzes these videos to determine the rodent's position at each time point. By tracking changes in position over time, the system provides detailed insights into rodent behavior, including speed, mobility, and climbing activity. To evaluate the system's reliability, we utilized a starvation-induced hyperactivity model, employed as a female mouse model for anorexia nervosa. This model is characterized by pronounced hyperactivity, typically assessed using electronically monitored running wheels. Both the Goblotrop system and running wheel measurements demonstrated that starvation increases food-anticipatory activity (up to 4 h before food availability) while reducing nocturnal activity. The results from the Goblotrop system and running wheel measurements exhibited remarkable consistency. Thus, the Goblotrop system proves to be a valuable tool for studying locomotor activity and circadian rhythms in different cage areas in animal models. This tool provides potential for various scientific fields, including neuroscience, pharmacology, toxicology, and behavioral research.

## Significance Statement

The activity and circadian rhythm of laboratory animals play a crucial role in neuroscience but are often difficult to measure. This paper introduces the Goblotrop system, a tool designed to monitor long-term changes in activity and circadian rhythm of laboratory animals throughout the day and night. The system analyses behavioral changes in mice subjected to food restriction.

## Introduction

Studying locomotor activity in animals is fundamental for understanding physiological and behavioral processes. It serves as a cornerstone in numerous scientific disciplines, including neuroscience, pharmacology, toxicology, behavioral metabolism, and health research. Locomotor activity, defined as the movement of an organism from one place to another, represents a key aspect of behavior. Analyzing locomotor activity in animal models provides valuable insights into how interventions, such as medications or genetic modifications, influence mobility. Beyond locomotion, behavioral studies in animals yield critical information about cognitive, emotional, and social functions. These include investigations into learning and memory processes, reward systems, and responses to stress. Such research is particularly impactful in understanding neurological disorders, mental health conditions, and other health-related behavioral effects.

**Figure 1.**
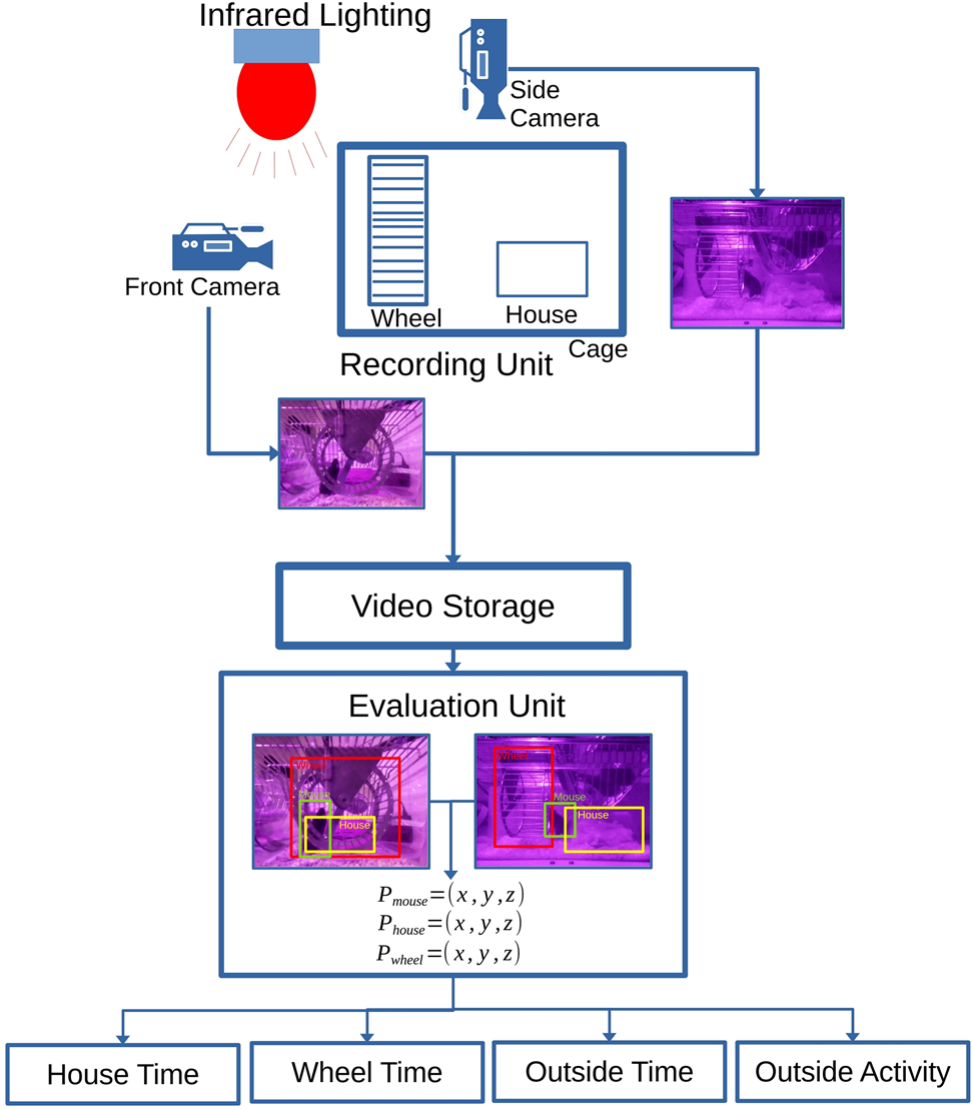
The figure illustrates the basic concept of the Goblotrop system, which consists of at least one recording unit. Each unit contains two infrared cameras that monitor a cage. The video data are transmitted to a video storage system, where an evaluation unit retrieves and analyzes the 3D positions of the mouse $\left(P_{\mathrm{mouse}}=(x,y,z)\right)$, house $\left(P_{\mathrm{house}}=(x,y,z)\right)$ and wheel $\left(P_{\mathrm{wheel}}=(x,y,z)\right)$. Using these positions, the evaluation unit calculates various parameters, including house time, wheel time, outside time, and outside activity.

We established the Goblotrop system, an artificial intelligence-based infrared sensor system, capable of localizing rodents within their laboratory environment, such as a cage. The system is designed to determine the subjects’ three-dimensional (3D) positional coordinates at specific temporal intervals. This function subsequently yields information about the time the rodents spent in distinct regions of the environment and enables detailed monitoring of their behavior and locomotion speed. In this context, the behavior is specified by the time the rodent spent in the house, in the running wheel, and outside of both these areas. As illustrated in Figure 1, the Goblotrop system utilizes at least (1) one recording unit, consisting of two infrared cameras and an (2) evaluation unit, and (3) infrared lighting. The cameras continuously capture video recordings of the environment, which are then analyzed using a specialized software for object detection, such as a convolutional neural network. This software extracts the rodent's two-dimensional (2D) position in each frame. Positions detected from two perspectives are subsequently combined to determine a 3D position. These 3D positions reveal the animal presence in specific areas of the environment, while changes in position over time provide data on their locomotion speed within the environment.

To analyze the reliability of the Goblotrop system, a mouse model known as starvation-induced hyperactivity (SIH) model is used to mimic the somatic symptoms of anorexia nervosa (AN; Frintrop et al., 2018a,b, 2019; Gabloffsky et al., 2022; Staffeld et al., 2023; Zimmermann et al., 2023). This eating disorder is associated with severe body weight loss, excessive locomotor activity, and amenorrhea. In the SIH model, mice were provided with limited food access along with running wheels. The food intake of the mice was reduced until they lost 25% of their initial body weight, a process referred to as the acute starvation phase, lasting 6 d. Following this, the body weight loss was maintained for an additional 2 weeks, mimicking chronic starvation, in the following referred to as chronic starvation phase. A previous study demonstrated that mice with a 20% reduction in body weight exhibit increased running activity, particularly during the 4 h before the animals were fed, a behavior known as food-anticipatory activity (FAA; Gabloffsky et al., 2022). This study aims to develop a robust tool for investigating behaviors, such as circadian rhythms and locomotor activity, in different parts of the cage in animal models.

### Relevant systems

The objective of this study requires adherence to specific criteria for the tracking system:

1. Rodents must remain within their standard cages, which are equipped with a top-mounted feeder and water dispenser.
2. Accurately determining the rodent's 3D position is essential for the system's effectiveness.
3. The system must be capable of operating continuously, ensuring functionality during both daytime and nighttime.

Current methodologies in the field provide various strategies for tracking rodent movement (Kahnau et al., 2023). A widely used approach involves a camera-based system that captures video recordings of the environment. These recordings are subsequently analyzed frame by frame using specialized algorithms to precisely determine the rodent's location (Tungtur et al., 2017; Singh et al., 2019; Krynitsky et al., 2020). An example of such a system is the “BioSense” system which employs a conventional camera (Patman et al., 2018). However, its detection algorithm requires a static background to identify the laboratory animal. Initially, the system applies a Gaussian mixture model for background subtraction, isolating the foreground subject (the rodent) from its background. The rodent's position is continuously tracked using a Kalman filter. Despite its capabilities, the system is limited by its inability to determine the rodent's 3D coordinates, rendering it unsuitable for our specific requirements.

Another system, “Live Mouse Tracker,” addresses this limitation by employing a red, green, and blue depth (RGBD) camera (de Chaumont et al., 2019). This RGBD camera can determine the distance of objects to the camera, similar to the one used by Rezaei et al. (2017). This camera captures standard video and depth data of its field of view. The depth data allow locating the rodents with an additional refinement through a machine learning technique, known as “random forest.” Beyond identifying the rodent's location, this system can distinguish between its head and tail. However, the system's effectiveness at night is questionable due to its reliance on infrared light for depth mapping. Additionally, it needs specialized housing.

Another open-source framework “PyRodentTracks” integrates a standard camera with multiple radio-frequency identification (RFID) sensors (Fong et al., 2022). Unlike the preceding systems, it uses a neural network (YoloV4) to determine the position of the rodents. While it could be adapted for night-vision capabilities, the manuscript does not explicitly address this feature.

In contrast to the video-based approaches, other systems evaluate the locomotor activity through running wheels, motion sensors, and/or beam breaks (Klein et al., 2022). Compared with running wheels, the Goblotrop system enables comprehensive tracking of locomotor behavior throughout the entire home cage, not just during wheel use. In contrast to RFID sensors, which require invasive attachment to the animal, Goblotrop provides noninvasive, continuous monitoring without disturbing natural behavior. Beam-break systems detect animal movement by registering interruptions in an infrared beam grid across the cage. Their spatial resolution depends on the density of this grid, but they cannot distinguish between animals and objects. In enriched environments, this can lead to missed detections and reduced data reliability. In contrast to beam-break systems, which lose accuracy in enriched environments, the Goblotrop system provides reliable high-resolution 3D tracking of animals, independent of objects present in the cage.

In summary, none of the mentioned systems fully meet all the requirements of our study, underscoring the need for further innovation in this field. Thus, this study aims to establish a tracking system that fulfills all three criteria. In addition, it systematically compares the Goblotrop system with the running wheel system called VitalView Activity Tracker.

## Materials and Methods

### The Goblotrop system

The Goblotrop system is designed to track rodents in their laboratory environment for at least 22 h, including transitions between light and dark phases. To achieve this, the system is composed of three main components: (1) one to four recording units, (2) an evaluation unit, and (3) infrared illumination. Figure 1 illustrates the structure of the Goblotrop system.

#### Recording of the videos

**Figure 2.**
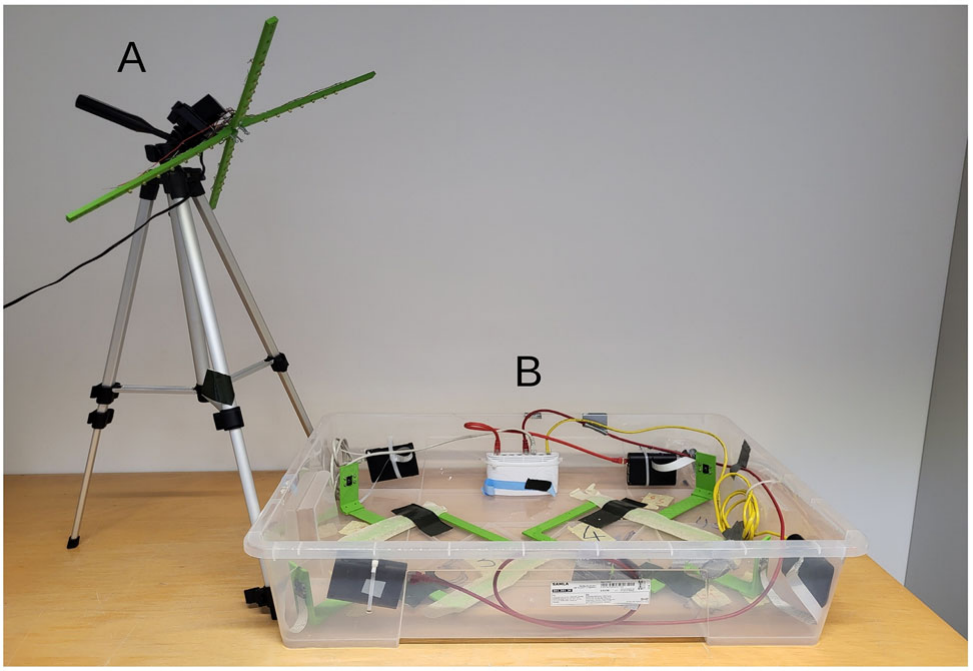
The figure shows the 3D-printed lamp with 28 IR-LEDs on a regular camera stand (***A***) next to two recording units (***B***).

Each recording unit consists of at least two infrared cameras. The cameras are mounted on a 3D-printed bracket. The mounting is designed for Eurostandard Type 2 (Tecniplast 1264C) cages. The cages used in this study are made of transparent plastic and measure up to 268 × 215 × 141 mm. The camera mounting is designed to accommodate a Raspberry Pi Camera Module 2 NoIR. The distance from the front camera to the cage is 100 mm. The distance of the side camera to the cage is 150 mm. Each Raspberry Pi Camera Module 2 NoIR is connected to a Raspberry Pi 3B+, which are further connected to a Raspberry Pi 4B via gigabit Ethernet. For the task at hand, a FritzBox 4020 and 2 gigabit switches serve as the backbone of the network. The Raspberry Pi 4B is connected to an external 8 TB hard drive, which serves as a network-attached storage (NAS). The NAS serves as a storage for eight video streams from the four recording stations, handling a total of eight video streams. Figure 2 shows the 3D-printed mount for the infrared illumination, which consists of 28 IR-LEDs. The illumination mount is attached to an off-the-shelf camera tripod.

#### Evaluation of the videos

The task of the evaluation unit is to analyze the videos regarding the following parameters:

1. House time: the time the rodent spent in the house
2. Wheel time: the time the rodent spent in the running wheel
3. Outside time: the time the rodent was neither in the house nor in the running wheel
4. Outside activity: the distance the rodent moved outside the house and the running wheel

The evaluation unit processes the videos and evaluates the position of the rodent, the house, and the wheel with a neural network called YoloV4 (Bochkovskiy et al., 2020). The video is divided into consecutive frames, each of which is processed through multiple stages of the neural network. The outputs of the YoloV4 network consist of multiple bounding boxes (BB), which are used to identify and track the rodent's position in each frame. A BB is characterized by a midpoint ((*x*,*y*)) of the identified object and the width and height of it: BB = (*x*,*y*,*w*,*h*). The network is trained to detect three objects: the rodent, the house, and the wheel. If the network fails to detect the mouse due to shadowing or untrained positions, the evaluation unit interpolates the position of the mouse across one or multiple frames. Based on the 2D positions of the rodent, the house and the wheel, the evaluation unit determines whether the rodent is inside the house $\left(BB_{h}\right)$ or the wheel $\left(BB_{w}\right)$. A conversion to calculate the wheel time only requires a change of the particular BB. The following equation checks whether the BB of the rodent *R* is inside the BB of, e.g., the house *H*:

$$\begin{array}{rl} & q^{\mathrm{front}}=(R_{x}^{\mathrm{front}}<(H_{x}^{\mathrm{front}}+0.5H_{w}^{\mathrm{front}})\\ & \wedge (R_{x}^{\mathrm{front}}<(H_{x}^{\mathrm{front}}-0.5H_{w}^{\mathrm{front}})\\ & \wedge (R_{y}^{\mathrm{front}}<(H_{y}^{\mathrm{front}}+0.5H_{h}^{\mathrm{front}})\\ & \wedge (R_{y}^{\mathrm{front}}<(H_{y}^{\mathrm{front}}-0.5H_{h}^{\mathrm{front}})\\ & q^{\mathrm{side}}=\ldots .\\ & q=q^{\mathrm{front}}\wedge q^{\mathrm{side}}. \end{array}$$

If Equation 1 becomes true for the front-perspective and the side-perspective, the rodent is counted inside the corresponding area for that particular frame. The total amount of time the rodent spent in the particular area is calculated with the following equation for the $t_{\mathrm{house}}$, with *n* defined as the total number of frames, FPS as the number of frames per second, and *f* as the number of the current frame:

$$t_{\mathrm{house}}=\;\frac{\sum_{0}^{n}\;q_{f}}{\mathrm{FPS}}.$$

However, with the timestamp of each frame and the positions, the evaluation unit calculates the movement of the rodent outside the wheel and the house. For this, the 2D positions are transformed to a 3D position $P=(x,y,z{)}^{T}$, resolving from the BB of the front (*F*) and from the side (*S*):

$$\begin{array}{rl} & P_{x}=F_{x}\\ & P_{y}=S_{x}\\ & P_{z}=0.5(F_{y}+S_{y}). \end{array}$$

The pixel distance *s* the rodent moved between two consecutive frames *f* in one perspective is calculated as follows:

$$\begin{array}{rl} & s=\;\sqrt{s_{x}+s_{y}+s_{z}}\\ & s_{x}=(P_{x}^{\left(\;f-1\right)}-P_{x}^{\left(f\right)}{)}^{2}\\ & s_{y}=(P_{y}^{\left(\;f-1\right)}-P_{y}^{\left(f\right)}{)}^{2}\\ & s_{z}=(P_{z}^{\left(\;f-1\right)}-P_{z}^{\left(f\right)}{)}^{2}. \end{array}$$

#### Software availability

In the sense of open source, we provide resources to construct and run a Goblotrop system. The resources contain SCAD scripts and STL files for 3D printing, scripts, and instructions for operating one or multiple cameras and scripts for the orchestration of the recording units. In addition, the resources contain scripts and guidelines for managing the evaluation unit. The resources are available at https://github.com/1tg137/Goblotrop.

#### Goblotrop setup and configurations

The prototypical implementation was designed to enable concurrent analysis of four rodents for at least 22 h. The resolution of the cameras was set to 1,640 × 1,232 pixels (px) with a frame rate of 15 FPS. The compression of the video streams was set to H.264. Tracking the 3D position of the rodents from two independent videos requires the videos to be synchronized. However, we did not expect a latency offset of 300 ms to have a significant impact on the task at hand. For detecting the mice in the videos, two YoloV4 networks were utilized. The front network was trained with a dataset that contains 20,271 hand-labeled frames taken by the front camera. The side network was trained with a dataset which contains 16,655 hand-labeled frames taken by the side camera. The training data were divided into a training set and a validation set. The training set consists of 80% of the training data. The validation set contained 20% of the dataset and consists of an equal distribution of video frames of the light and the dark phase. The video evaluations were conducted on a GPU server operating with Ubuntu 22.04, which leverages four NVIDIA RTX 2080Ti graphics cards to perform computations. The networks were adjusted to fit the maximum GPU memory available on the RTX 2080Ti. The input size of the network was set to 832 × 608. The networks were validated with the 0.5 mAp metric. Mean average precision (mAP): The front network achieved precision scores of 78% for the mouse, 83% for the wheel, and 97% for the house. The side network achieved the following precision: mouse, 98%; house, 99%; and wheel, 99%. To ensure the correct functioning of the entire system, we verified the frame-wise duration of the mouse's presence inside the house and the wheel in the recorded videos.

#### Animals, study design, and locomotor activity determination

The 4-week-old female C57BL/6J mice purchased from Janvier Labs are part of a large cohort study (Staffeld et al., 2023) and were maintained under a 12/12 h light/dark cycle (lights on at 6 A.M.) with controlled temperature of 22 ± 2°C. Cages were changed once a week, and microbiological monitoring was performed according to the Federation of European Laboratory Animal Science Associations recommendations. All experimental procedures were approved by the Review Boards for the Care of Animal Subjects of the district government of Mecklenburg-Western Pomerania (reference number 7221.3-1-005/21). The induction of the SIH model has been previously described (Gabloffsky et al., 2022; Staffeld et al., 2023). In brief, after an acclimatization phase of 10 d with *ad libitum* access to food and water and daily animal handling, the mice were randomly assigned to different treatment groups on the first experimental day. Throughout the experiment, the body weight and food consumption (measured daily by weighing the food) were recorded daily at 1 P.M. At this time, the feeding was performed in the acute and chronic starvation phase. Wheel running was measured using an exercise wheel (11.5 cm in diameter) mounted on the top of a standard mouse cage. The revolutions of the running wheel were monitored and summed every hour with an activity software (VitalView Activity 1.4, STARR Life Science). The acute starvation phase included 1 week of starvation, during which the mice received 40% of their average daily food intake until a 25% body weight loss was achieved. Once this body weight loss was reached, the daily food intake was adjusted to maintain the 25% weight loss. To mimic chronic starvation, the acute starvation phase was followed by additional 2 weeks of starvation (control, *n* = 5; SIH, *n* = 9). In this phase, the mice received 45–70% of their average daily food intake. If the body weight differed by >2.5% from the target weight, the provided food amount was adjusted in increments of 5%. The control groups were housed under the identical conditions but had *ad libitum* access to food during the whole experiment. The Goblotrop system examined the mice at the end of each phase.

Each animal was housed individually in a cage with a running wheel purchased from STARR Life Science. The running wheel activity (RWA) was monitored with an activity software (VitalView Activity 1.4, STARR Life Science) using a digital magnetic counter which was attached to the wheel and connected to a microprocessor that stored the number of revolutions per hour. For analysis of the RWA, different periods were defined: FAA (4 h, from 9 A.M. to 1 P.M.), postprandial activity (PA; 4 h, from 2 P.M. to 6 P.M.), night activity (NA; 12 h, 6 P.M. to 6 A.M. next day), and preprandial activity (PRA; 3 h, from 6 A.M. to 9 A.M.). The feeding time (1 P.M. to 2 P.M.) was excluded from the analysis. The VitalView Activity system delivers the sum of rotations *R* of the wheel per hour. In combination with the wheel time $t_{\mathrm{wheel}}$, the Goblotrop system calculates the running speed $v_{\mathrm{wheel}}$ of the mice inside the wheel:

$$v_{\mathrm{wheel}}=\;\frac{R}{t_{\mathrm{wheel}}}.$$

#### Statistics

Data are represented as means and standard errors of the mean. For statistical testing, the values for RWA were compared for the acclimatization phase (Days 1–10), the acute starvation phase (Days 11–16), and the chronic starvation phase (Days 17–29). The comparisons of the parameters of RWA in the different periods (FAA, PA, NA, PRA) between SIH mice and controls within each phase of starvation were evaluated by two-way ANOVA with repeated measurements with a significance level of 5%. The parameters wheel time, house time, outside time, outside activity, running speed, and revolutions were also analyzed with two-way ANOVA with repeated measurements, with a significance level of 5%. The correlation between wheel time and the RWA was calculated using Pearson's correlation. All the above analyses were conducted using SPSS version 20 for Windows (IBM). Table 1 summarizes all significant statistics.

**Table 1.** The table presents the various statistical findings regarding the different phases of the study

Fig.	Graph	Data structure	Type of test	*p* values	Power
3	B—acute	ND	Two-way ANOVA with RM	≤0.001	*η*^2^ = 0.573
3	B—chronic	ND	Two-way ANOVA with RM	≤0.001	*η*^2^ = 0.631
3	C—chronic	ND	Two-way ANOVA with RM	≤0.001	*η*^2^ = 0.618
3	D—chronic	ND	Two-way ANOVA with RM	≤0.001	*η*^2^ = 0.924
3	E—chronic	ND	Two-way ANOVA with RM	≤0.01	*η*^2^ = 0.512
4	A—acute Light Phase 1	ND	Two-way ANOVA with RM	≤0.01	*η*^2^ = 0.458
4	A—acute Light Phase 2	ND	Two-way ANOVA with RM	≤0.01	*η*^2^ = 0.510
4	A—chronic Light Phase 2	ND	Two-way ANOVA with RM	≤0.01	*η*^2^ = 0.485
4	B—acute Dark Phase	ND	Two-way ANOVA with RM	≤0.05	*η*^2^ = 0.405
4	B—acute Light Phase 2	ND	Two-way ANOVA with RM	≤0.05	*η*^2^ = 0.295
4	B—chronic Dark Phase	ND	Two-way ANOVA with RM	≤0.05	*η*^2^ = 0.391
4	C—chronic Light Phase 1	ND	Two-way ANOVA with RM	≤0.05	*η*^2^ = 0.270
4	C—chronic Dark Phase	ND	Two-way ANOVA with RM	≤0.01	*η*^2^ = 0.550
4	D—acute Dark Phase	ND	Two-way ANOVA with RM	≤0.05	*η*^2^ = 0.360
4	E—acute Dark Phase	ND	Two-way ANOVA with RM	≤0.01	*η*^2^ = 0.437
4	E—acute Light Phase 2	ND	Two-way ANOVA with RM	≤0.01	*η*^2^ = 0.478
4	E—chronic Light Phase 1	ND	Two-way ANOVA with RM	≤0.05	*η*^2^ = 0.290
4	E—chronic Light Phase 2	ND	Two-way ANOVA with RM	≤0.05	*η*^2^ = 0.386
4	F—acute Light Phase 2	ND	Two-way ANOVA with RM	≤0.01	*η*^2^ = 0.528
4	F—chronic Light Phase 2	ND	Two-way ANOVA with RM	≤0.001	*η*^2^ = 0.730
6		Non-ND	Pearson's correlation	≤0.001	*η*^2^ = 0.793

ND, normal distributed; RM, repeated measures.

## Results

### Acute and chronic starvation lead to an increase in FAA

The findings regarding body weight and 24 h running activity are reported in the study by Staffeld et al. (2023). To analyze RWA measured with running wheels for different daily periods of the mice, the following phases were predefined: FAA (4 h, from 9 A.M. to 1 P.M.), PA (4 h, from 2 P.M. to 6 P.M.), NA (12 h, 6 P.M. to 6 A.M. next day), and PRA (3 h, from 6 A.M. to 9 A.M.).

**Figure 3.**
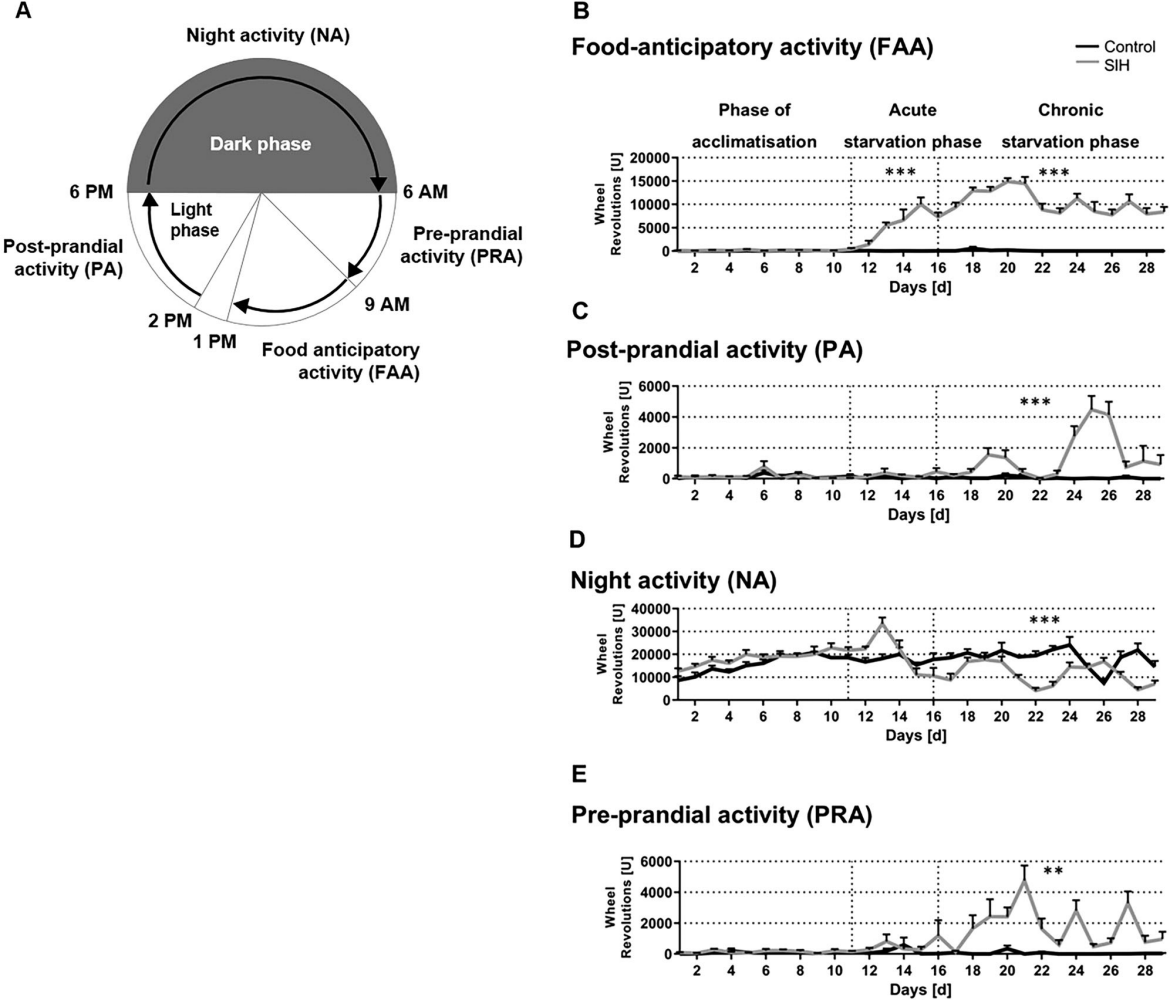
The figure shows (***A***) the experimental structure of different daily periods of running activity of cycles of 24 h and (***B–E***) the daily revolutions of the VitalView running wheels over different phases of our study. Acute and chronic starvation leads to an increase in FAA. ***B***, The FAA (4 h, from 10 A.M. to 1 P.M.), (***C***) the PA (4 h, from 2 P.M. to 6 P.M.), (***D***) the NA (12 h, 6 P.M. to 6 A.M. the next day), and (***E***) the PRA (4 h, 6 A.M. to 10 A.M.) daily periods during acute and chronic starvation in SIH mice are investigated using running wheel sensors. **p ≤ 0.01; ***p ≤ 0.001; two-way ANOVA with repeated measurements.

During the phase of acclimatization, no differences in FAA were detectable between the analyzed groups. Acute and chronic starvation led to an increase in FAA of SIH mice (Fig. 3*B*, acute starvation phase, control, 103 *U* ± 24; SIH, 6,683 *U* ± 1,059; *p* ≤ 0.001; chronic starvation phase, control, 85 *U* ± 78 vs SIH, 10,554 *U* ± 1,172; *p* ≤ 0.001). Thus, chronic starvation induced an increase in FAA. During the phase of acclimatization and acute starvation, no differences in PA were detectable between the analyzed groups. Chronic starvation led to an increase in PA in SIH mice (Fig. 3*C*; control, 55 *U* ± 44; SIH, 1,521 *U* ± 496; *p* ≤ 0.001). No differences in NA were detected among the examined groups throughout the acclimatization phase and during the acute starvation phase. Chronic starvation led to a decrease of NA in SIH mice (Fig. 3*D*; control, 18,583 *U* ± 2,153; SIH, 11,624 *U* ± 1,587; *p* ≤ 0.001). No differences in PRA were detectable between the analyzed groups during the phase of acclimatization and acute starvation. Chronic starvation led to an increase in PRA in the SIH mice (Fig. 3*E*; control, 48 *U* ± 32; SIH, 1,876 *U* ± 608; *p* ≤ 0.01).

### The Goblotrop system validates the increase in FAA

**Figure 4.**
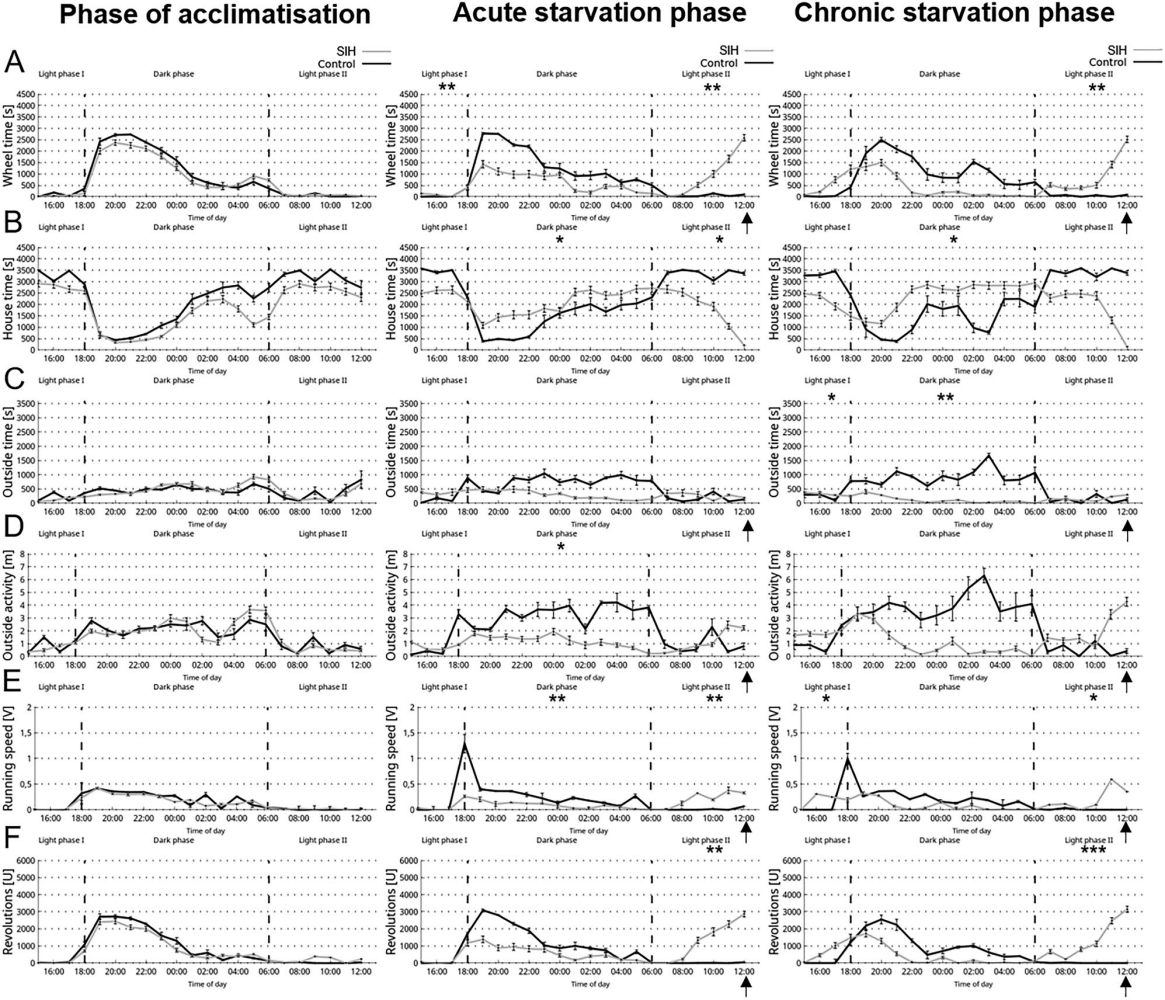
Starvation leads to decreased circadian rhythm-related activity at night. The Goblotrop system measured various parameters, including (***A***) wheel time, (***B***) house time, (***C***) outside time, and (***D***) outside activity. The (***F***) revolutions of the running wheel was measured with the VitalView Activity system and (***E***) were calculated with ***A*** and ***F*** using Equation 5. The parameters were measured during acclimatization, acute starvation, and chronic starvation in SIH (*n* = 9) and corresponding control mice (*n* = 5). Daily at 1 P.M., food was provided to the mice (marked with arrows). *p ≤ 0.05, **p ≤ 0.01, ***p ≤ 0.001; two-way ANOVA with repeated measurements.

Figure 4 visualizes the following parameters: wheel time, house time, outside time, outside activity, revolutions of the wheel, and the running speed. The first four parameters were measured with the Goblotrop system, while the revolutions were measured with the VitalView Activity system. The results are segmented into three different phases: Light Phase 1 (3 P.M. to 6 P.M.), Dark Phase (6 P.M. to 6 A.M.), and the Light Phase 2 (6 A.M. to 1 P.M.). During acute and chronic starvation, the wheel time measured by the Goblotrop system of SIH mice was increased during Light Phase 2, which included the time of FAA and of PRA (Fig. 4*A*; acute starvation phase, control, 343 s ± 108; SIH, 5,883 s ± 597; *p* ≤ 0.01; chronic starvation phase, control, 279 s ± 133; SIH, 5,666 s ± 648; *p* ≤ 0.01). In addition, during acute and chronic starvation, the revolutions of the running wheel in SIH mice increased during Light Phase 2 (Fig. 4*F*; acute starvation phase, control, 99 *U* ± 26; SIH, 6,403 *U* ± 851; *p* ≤ 0.01; chronic starvation phase, control, 43 *U* ± 15; SIH, 6,511 *U* ± 812; *p* ≤ 0.001). In summary, starvation induced an increase in FAA which was measured with both systems (Goblotrop and VitalView Activity system).

### Starvation leads to a decreased nocturnal activity measured with the Goblotrop system

During the acclimatization phase, no significant differences were observed in any of the analyzed parameters between the SIH and control groups. In the Dark Phase, starvation induced an increase in the house time in SIH mice in comparison with control mice (Fig. 4*B*; acute starvation phase, control, 16,627 s ± 2,011; SIH, 24,523 s ± 1,999; *p* ≤ 0.05; chronic starvation phase, control, 16,521 s ± 2,948; SIH, 29,339 s ± 1,930; *p* ≤ 0.05). In Light Phase 2 of the acute starvation phase, the SIH-mice showed an decrease in the house time (Fig. 4*B*; control, 20,259 s ± 417; SIH, 10,497 s ± 868; *p* ≤ 0.05). During chronic starvation, the outside time in SIH mice was decreased in Light Phase 1 (Fig. 4*C*; chronic starvation phase, control, 1,529 s ± 104; SIH, 1,263 s ± 307; *p* ≤ 0.05) and decreased in the Dark Phase (chronic starvation phase, control, 11,366 s ± 1,589; SIH, 1,353 s ± 242; *p* ≤ 0.01). During acute starvation, the outside activity decreased in the Dark Phase (Fig. 4*C*, acute starvation phase, control, 40 m ± 4; SIH, 14 m ± 2; *p* ≤ 0.05). During acute starvation, the SIH mice demonstrated a decrease in the running speed in the Dark Phase (Fig. 4*E*; acute starvation phase, control, 2.69 m/s ± 0.39; SIH, 0.99 m/s ± 0.20; *p* ≤ 0.01) and an increase in Light Phase 2 (acute starvation phase, control, 0.08 m/s ± 0.01; SIH, 1.36 m/s ± 0.19; *p* ≤ 0.01). During chronic starvation, the SIH mice showed an increase in the running speed in Light Phase 1 (Fig. 4*E*; chronic starvation, control, 0.99 m/s ± 0.1; SIH, 0.75 m/s ± 0.04; *p* ≤ 0.05) and in Light Phase 2 (chronic starvation, control, 0.04 m/s ± 0.02; SIH, 1.36 m/s ± 0.03; *p* ≤ 0.05). In brief, both acute and chronic starvation led to alterations in activity patterns during both dark and light phases.

### The wheel time measured with the Goblotrop system is associated with the revolutions analyzed by the VitalView Activity system

**Figure 5.**
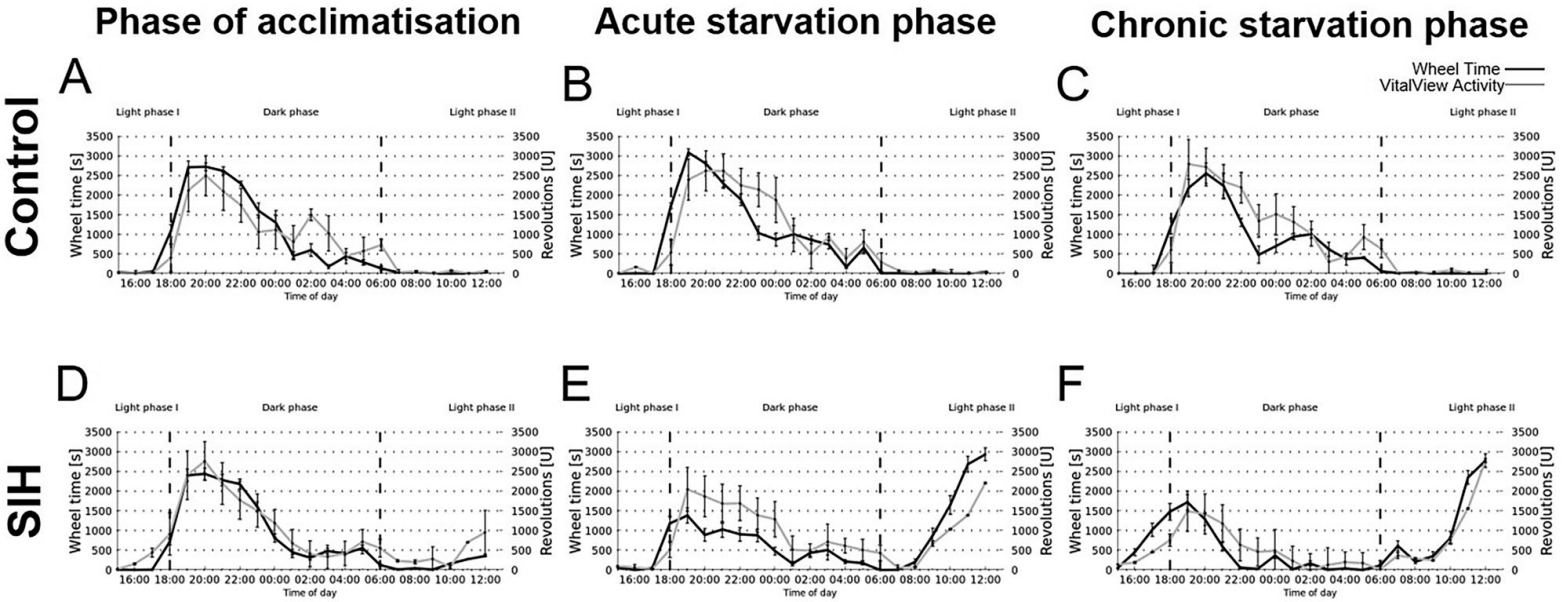
The wheel time measured by the Goblotrop and the revolutions measured by the VitalView Activity running wheels show a similar progression regarding the minimum and maximum during the three starvation phases. The figure shows the average wheel time (black), measured by the Goblotrop and the average revolutions of the VitalView Activity running wheel (gray) for the SIH (***A–C***) and the control animals (***D–F***) during the three phases of the experiment.

**Figure 6.**
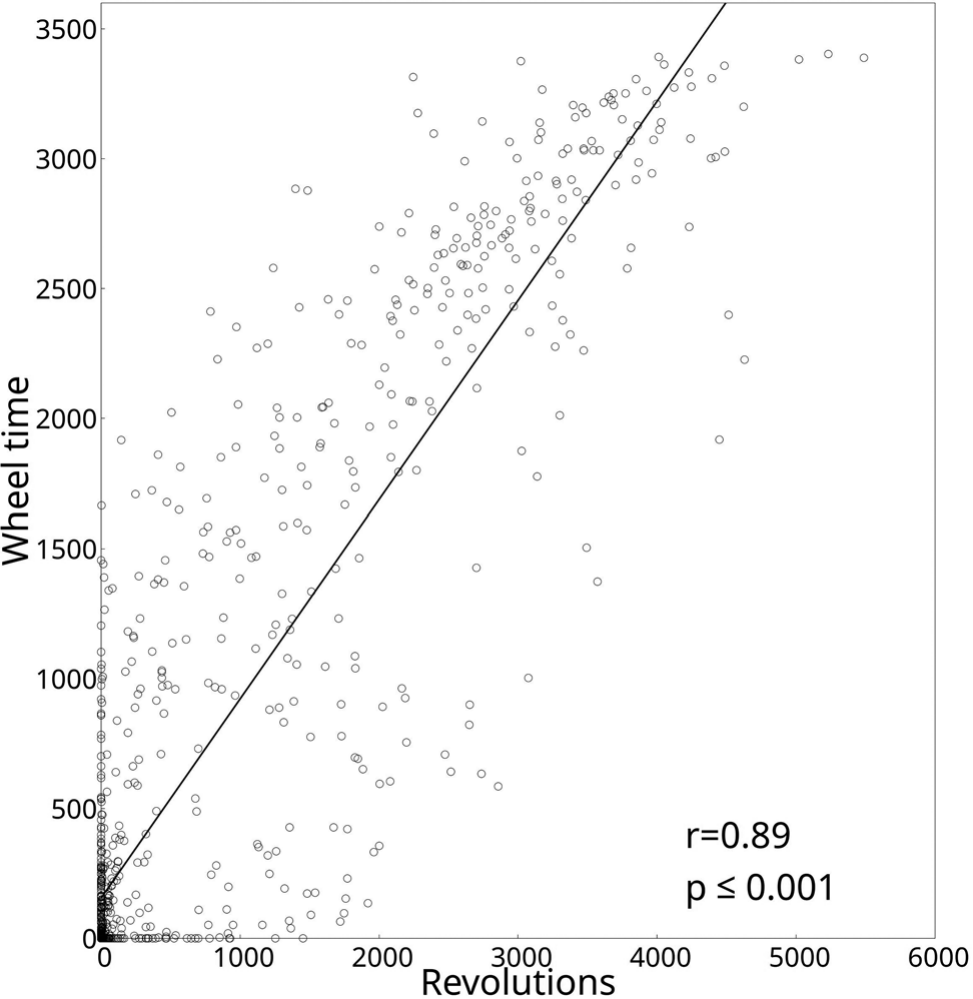
The figure illustrates the relationship between the wheel time and the revolutions measured by the VitalView Activity running wheel, based on 902 data points of control and SIH animals (in all phases). The data show a Pearson's correlation coefficient of *r* = 0.89 with a significance level of *p* ≤ 0.001.

Figure 5 illustrates the wheel time measured with the Goblotrop and the revolutions of the VitalView Activity running wheel. The diagrams show the same trend of the time spend in the wheel and the measured revolutions, e.g., the maximum or minimum of the activity is detected by both systems at the same time. Figure 6 shows the wheel time measured by the Goblotrop and the revolutions measured by the VitalView running wheel of all control and SIH mice during all phases (*n* = 902). The data show a Pearson's correlation coefficient of *p* = 0.89 with a significance level of *p* ≤ 0.001.

## Discussion

We developed the Goblotrop system, an open-source camera setup for analyzing rodent locomotor behavior in lab environments. It includes at least two cameras, infrared lighting, a neural network for tracking positions, and software for behavior analysis. The system was used to study mouse behavior in the SIH model across three phases with varying food availability. Results from Goblotrop aligned with data from traditional monitoring via the VitalView Activity system. The data, as shown in Figure 6, show a highly significant correlation (*r* = 0.89; *p* < 0.001) between the revolutions of the VitalView running wheel and the wheel time measured by the Goblotrop system. These findings highlight the Goblotrop system as a valuable tool in the study of rodent activities, because the recorded videos can be reanalyzed with different approaches. However, the difference in the measured rotations of the VitalView running wheel and the Goblotrop system highlights the differences between these two tools of measurement: the running wheel is only able to measure the rotations whereas the Goblotrop system measures the time inside the running wheel. However, from a technical perspective, the Goblotrop system has the potential to directly measure the rotations of the running wheel. In summary, our results show that both systems detected similar activities of the mice during the different phases of starvation. Furthermore, the Goblotrop system delivered additional parameters for characterizing the behavior of the mice.

The reliability of the study was confirmed by several findings:

1. Starvation induced an increase in FAA shown with both measurements.
2. Starvation decreased nocturnal activity in SIH mice at night.
3. The increase in FAA and nocturnal activity changes were previously shown in animal models for AN (Lewis and Brett, 2010; Beeler et al., 2021).

Analyses by chronobiologists indicate that FAA is controlled by a circadian clock distinct from the light-entrained clock regulating general activity rhythms (Mistlberger, 2009, 2011). Therefore, circadian locomotor changes observed in starved mice may be driven by an increase in FAA. In a previous study, we showed that chronic starvation induces circadian alterations, assessed by cosinor analysis, which are associated with glial changes in the hypothalamus (Zimmermann et al., 2025). Accordingly, future studies will investigate whether these circadian changes depend on the timing of FAA.

A drawback of the Goblotrop system is the large volume of video data—990 h totaling around 10 TB. Like all tracking systems, it requires significant computational resources, particularly for neural networks used in object detection. Goblotrop uses YOLOv4 networks, offering a good balance between speed and accuracy: 96.0% detection from the front view and 99.87% from the side, averaging 97.98% overall. Missing single-frame detections are interpolated using the last known position. This led to 26.31 s of undetected rotations (1.7% of those measured by the VitalView system). Accuracy could be improved by adding more training data from missed detections. In general, the system demonstrated robustness to minor changes in the environment, such as slight adjustments to the camera position or the presence of laboratory personnel in the background. However, more substantial modifications to the setup may require retraining the network with additional training data. Such changes include, for example, alterations in the color of the house or cage or the addition of new objects to the cage.

To manage the computational workload of our task, we employed multiple instances of consumer-grade graphic processing units (GPU) RTX 2080Ti. A single RTX2080Ti GPU can evaluate two videos at a steady frame rate of 25 FPS. The GPU achieves a frame rate of 13 FPS for four videos processed in parallel. With a slightly reduced frame rate of the videos, an RTX 2080Ti GPU can do an online analysis of two rodents. It is noteworthy that more modern counterparts have surpassed these GPUs, e.g., RTX 4060TI, while their approximate cost remains the same. The power consumption of such a GPU lies ∼200 W.

Recently, the iMouse System was introduced as a tracking solution that also fulfills our three main criteria: rodents remain in their home cages, the system accurately determines their 3D position, and it functions reliably under both light and dark conditions (Laz et al., 2023). By comparison, the Goblotrop system stands out for its notably low cost, making it a more accessible option. Furthermore, the Goblotrop system enables detailed quantification of behaviors relevant to SIH—such as rearing, circadian activity shifts, sleep–wake patterns, grooming, and object interaction—directly within the home cage. This noninvasive approach minimizes stress by avoiding handling or novel environments, making it ideal for chronic behavioral phenotyping in models of anorexia and metabolic disorders.

In conclusion, the Goblotrop system proves to be a valuable tool for studying rodent behavior in laboratory settings, successfully meeting all three evaluation criteria. While its computational demands are considerable, ongoing advancements in image processing and hardware are likely to reduce associated costs over time. Currently, operating the system requires specialized expertise; however, future development efforts will aim to streamline its use, making it accessible to users without technical backgrounds.

## Synthesis

Reviewing Editor: Charlotte Cornil, Universite de Liege Decisions are customarily a result of the Reviewing Editor and the peer reviewers coming together and discussing their recommendations until a consensus is reached. When revisions are invited, a fact-based synthesis statement explaining their decision and outlining what is needed to prepare a revision will be listed below. The following reviewer(s) agreed to reveal their identity: Guillaume Drion.

This manuscript has now been reviewed by two experts who both agree that the proposed approach is interesting and brings some improvements compared to the conventional method. However, they raise important methodological issues, in particular the lack of validation of AI-based tracking compared to human-based tracking performance, that need to be addressed. They also question the benefit of this approach compared to the methods classically used to quantify home cage activity. More work is thus needed to provide missing validations and convince about the benefits of the proposed methods over the classic one.

Reviewer comments

Reviewer 1

The paper proposes an infrared-based method to track the 3D position of a rodent in its cage during experiments. The main improvement as compared to the state-of-the-art is the usability in both light and dark environments, which is a limitation of classical camera-based methods. The method uses convolutional neural networks for automatic tracking of the rodent and its surroundings (house, running wheel), and the proposed software is open source. The method is then validated experimentally on a starvation-induced hyperactivity rodent model which is characterized by pronounced hyperactivity and compared with running wheel measurements.

I find this method of great interest for the experimental community, but I have one main concern regarding the neural network part that I feel must be addressed to make the paper suitable for publication. In particular, there is an absence of metrics quantifying the tracking performance of the trained Neural Network. It is mentioned that "The 'front' network was trained with 40,000 hand-labeled frames taken by the front-camera. The 'side' network was trained with 29,000 hand-labeled frames taken by the side-camera". Were all the hand-labeled frames used as the training set, or were some of them used as a test set? If the authors used a test set to assess the training performance, the results should be made available in the paper. If not, it should be done, because there would be no scientific measure of the tracking accuracy of the system otherwise.

In addition, it would be interesting to study the robustness of the approach to slight changes in camera positions, which could occur during successive experiments. Would the authors suggest to retrain the networks each time the set-up configuration I slightly modified, or is there a measured tolerance zone in which the set-up could be adapted without retraining?

Reviewer 2

This manuscript describes an AI-based tracking system for quantifying locomotor activity in single housed mice in standard laboratory caging. The components and capabilities of the system are adequately specified and illustrated, and a validation is provided by comparing 'time in running wheel' derived from the neural network analysis with wheel revolutions collected by a commercially available data acquisition system. The correspondence between 'time in wheel' and 'number of wheel revolutions' detected by Vitalview, illustrated in Figure 5, is striking, and indicates little or no change in running speed across the night.

For additional validation, I would have liked to have seen a comparison of the AI data of 'time in wheel' or 'time in house' with data based on human visual scoring of the videos, which would be the gold standard. If the authors have such data (which I assume they would have collected to confirm that the algorithm works as expected), it would be worth including. If this has already been established in prior validation studies, this could be stated in the description of the algorithm.

While the manuscript does demonstrate the utility of this system for reproducing activity levels and patterns routinely recorded using running wheels, motion sensors and/or beam-breaks, the advantages of the video system over these others is not discussed. What additional elements of behaviour relevant to 'starvation induced hyperactivity' can this system quantify that would make it worth adopting?

It is interesting how the study of food restriction and locomotor activity are conducted in parallel within two largely separate fields of inquiry, one interested in modeling anorexia in humans, the other ('chronobiology') in understanding timing mechanisms controlling daily rhythms. Analyses of FAA conducted by chronobiologists indicates that it is controlled in part by a circadian clock, separate from the clock that controls activity rhythms synchronized by light dark cycles. When food is restricted to limited amount provided at a fixed time in the light period, circadian clock-controlled FAA emerges within a few days, while nocturnal activity typically decreases (depending on the type of activity being measured). The decrease in nocturnal activity does not appear to reflect changes in the phase or amplitude of the light-dark entrained circadian clock. For these reasons, it is preferable to refer to nocturnal activity as 'nocturnal activity', rather than as 'circadian-rhythm-related activity at night'. Chronobiological study of FAA is quite extensive, and the literature does include a number of methods papers, as well as studies that have employed video-based image analysis to quantify FAA

## Author Response

Dear Editors, Dear Reviewers, We thank you for reviewing our manuscript and providing helpful comments and suggestions. We have revised the manuscript accordingly and marked all changes in the new Word document. Additions are highlighted in bold font.

Reviewer comments Reviewer 1 The paper proposes an infrared-based method to track the 3D position of a rodent in its cage during experiments. The main improvement as compared to the state-of-the-art is the usability in both light and dark environments, which is a limitation of classical camera-based methods. The method uses convolutional neural networks for automatic tracking of the rodent and its surroundings (house, running wheel), and the proposed software is open source. The method is then validated experimentally on a starvation-induced hyperactivity rodent model which is characterized by pronounced hyperactivity and compared with running wheel measurements.

Question 1:

In particular, there is an absence of metrics quantifying the tracking performance of the trained Neural Network. It is mentioned that "The 'front' network was trained with 40,000 hand-labeled frames taken by the front-camera. The 'side' network was trained with 29,000 hand-labeled frames taken by the side-camera". Were all the hand-labeled frames used as the training set, or were some of them used as a test set? If the authors used a test set to assess the training performance, the results should be made available in the paper. If not, it should be done, because there would be no scientific measure of the tracking accuracy of the system otherwise.

Answer 1:

Thank you for this comment. The training data were divided into a training set (80 % of the data) and a validation set (20 %). In addition, we ensured that the validation data had equal images from the light and dark phases. To allow a better comparison of the used neural network, we have implemented the results of the network with the 0.5 mean average precision mAP-metric into the Section 2.1.4. of the paper:

2 Materials and Methods, 2.1.4 Goblotrop Setup and Configurations: "The front network achieved a mean Average Precision (mAP) of 78 % for the mouse, 83 % for the wheel, and 97 % for the house. The side network achieved the following precision: mouse: 98 %; house: 99 %; wheel: 99 %. To ensure the correct functioning of the entire system, we verified the frame-wise duration of the mouse's presence inside the house and the wheel in the recorded videos." Question 2:

In addition, it would be interesting to study the robustness of the approach to slight changes in camera positions, which could occur during successive experiments. Would the authors suggest to retrain the networks each time the set-up configuration I slightly modified, or is there a measured tolerance zone in which the set-up could be adapted without retraining? Answer 2:

The question is highly relevant when deploying the system in a new environment. For the initial experimental series, it may be necessary to retrain the network to adapt to the new conditions. Once trained, however, the network is expected to be robust to minor variations in the setup, such as slight shifts in camera position. More substantial changes-such as using mice with white instead of black fur-would, however, require retraining. Currently, there is neither a defined tolerance range nor an estimate of how such changes affect system accuracy. We have now set up the system multiple times and were able to accommodate such minor changes with minimal effort. We address this issue in Section 4 of the paper.

4 Discussion: "In general, the system demonstrated robustness to minor changes in the environment, such as slight adjustments to the camera position or the presence of laboratory personnel in the background. However, more substantial modifications to the setup may require retraining the network with additional training data. Such changes include, for example, alterations in the color of the house or cage, or the addition of new objects to the cage." Reviewer 2 This manuscript describes an AI-based tracking system for quantifying locomotor activity in single housed mice in standard laboratory caging. The components and capabilities of the system are adequately specified and illustrated, and a validation is provided by comparing 'time in running wheel' derived from the neural network analysis with wheel revolutions collected by a commercially available data acquisition system. The correspondence between 'time in wheel' and 'number of wheel revolutions' detected by Vitalview, illustrated in Figure 5, is striking, and indicates little or no change in running speed across the night.

Question 1: For additional validation, I would have liked to have seen a comparison of the AI data of 'time in wheel' or 'time in house' with data based on human visual scoring of the videos, which would be the gold standard. If the authors have such data (which I assume they would have collected to confirm that the algorithm works as expected), it would be worth including. If this has already been established in prior validation studies, this could be stated in the description of the algorithm.

Answer 1:

Thank you for this comment. The correct functioning of the system is ensured through multiple independent verification procedures (see also Question 1 of Reviewer 1). In the first step, we used a large bulk of hand-labeled training data to train and validate the network (80% of the data for training, 20% of the data for validation). This ensures that the detection of the neural network works properly. This step is a good measure for the comparison to a human measurement, because the subsequent analysis is only a logical conjunction of the positions measured by the neural network. We added a comparison of the visual capabilities of the network in comparison to a human via the 0.5 mAP-Metric in Section 2.1.4. The second step in the verification process involves validating the time-counting mechanism. We ensured the system's correct functioning by counting the frames during which the mouse stayed inside the house and on the wheel in test videos.

2 Materials and Methods, 2.1.4 Goblotrop Setup and Configurations: "The front network achieved a mean Average Precision (mAP) of 78 % for the mouse, 83 % for the wheel, and 97 % for the house. The side network achieved the following precision: mouse: 98 %; house: 99 %; wheel: 99 %. To ensure the correct functioning of the entire system, we verified the frame-wise duration of the mouse's presence inside the house and the wheel in the recorded videos." Question 2:

While the manuscript does demonstrate the utility of this system for reproducing activity levels and patterns routinely recorded using running wheels, motion sensors and/or beam-breaks, the advantages of the video system over these others is not discussed.

Answer 2: Thanks for this note. The advantages of the Goblotrop system compared to other methods, such as running wheels, lie in its ability to record the full 3D position of mice within their home cage. With running wheels, only locomotor behavior inside the wheel can be measured, making it impossible to infer activity patterns in other areas of the cage. For instance, when a mouse is inside the house, this may be a hint of sleep or rest behavior. In contrast to motion sensors, which require fixation on or implantation in the animal-making them invasive and impractical-the Goblotrop system is entirely non-invasive. An activity tracking system based on beam breaks operates by installing a grid of infrared beams across the x, y, and z axes of the cage. The system determines the position of the mouse based on the interruption of these beams. Consequently, the spatial resolution and accuracy depend heavily on the density of the beam grid. A key limitation of this approach lies in its detection principle: the system cannot distinguish between the mouse and other objects placed within the cage. Moreover, if the mouse moves behind such an object, it may not be detected at all. This significantly reduces the reliability of the data in enriched or complex environments.

In comparison, our video-based tracking system offers higher spatial precision and consistent detection regardless of cage complexity. It is unaffected by additional objects or structural enrichments, and it can reliably differentiate and follow the animal's position at all times. In summary, the Goblotrop system provides a robust, non-invasive, and high-resolution method for tracking the locomotor behavior of rodents in a home cage environment, even under complex and enriched conditions.

4 Discussion: "In contrast to the video-based approaches, other systems evaluate the locomotor activity through running wheels, motion sensors and/or beam breaks [1]. Compared to running wheels, the Goblotrop system enables comprehensive tracking of locomotor behavior throughout the entire home cage, not just during wheel use. In contrast to motion sensors, which require invasive attachment to the animal, Goblotrop provides non-invasive, continuous monitoring without disturbing natural behavior. Beam-break systems detect animal movement by registering interruptions in an infrared beam grid across the cage. Their spatial resolution depends on the density of this grid, but they cannot distinguish between animals and objects. In enriched environments, this can lead to missed detections and reduced data reliability. In contrast to beam-break systems, which lose accuracy in enriched environments, the Goblotrop system provides reliable high-resolution 3D tracking of animals, independent of objects present in the cage." Question 3:

What additional elements of behaviour relevant to 'starvation induced hyperactivity' can this system quantify that would make it worth adopting? Answer 3:

The Goblotrop system offers several advantages for studying behavioral elements relevant to starvation-induced hyperactivity beyond standard locomotor tracking. In particular, the following behavioral parameters can be quantified: • Rearing behavior - an important marker of exploratory drive and arousal, which often increases in food-restricted animals. • Circadian activity patterns - precise analysis of activity distribution across light-dark cycles, helping to detect shifts in the circadian rhythm commonly observed under restricted feeding. • Sleep-wake behavior - periods of rest versus activity can be inferred, providing insights into sleep fragmentation or sleep loss, which are often part of the hyperactivity phenotype. • Grooming and head-raising - with retraining of the neural network, these behaviors can be detected, offering information about stress levels and arousal states. • Interaction with objects or nesting material - behavioral tasks such as nest building or novel object recognition can be integrated to assess cognitive or affective changes under starvation.

A major strength of the system is that all measurements are performed in the home cage without requiring handling or transfer to a novel environment, thus minimizing stress. This makes the Goblotrop setup particularly well-suited for chronic studies and behavioral phenotyping in models of anorexia or other metabolic disorders.

4 Discussion: "Further, the Goblotrop system enables detailed quantification of behaviors relevant to starvation-induced hyperactivity-such as rearing, circadian activity shifts, sleep-wake patterns, grooming, and object interaction-directly within the home cage. This non-invasive approach minimizes stress by avoiding handling or novel environments, making it ideal for chronic behavioral phenotyping in models of anorexia and metabolic disorders." Question 4:

It is interesting how the study of food restriction and locomotor activity are conducted in parallel within two largely separate fields of inquiry, one interested in modeling anorexia in humans, the other ('chronobiology') in understanding timing mechanisms controlling daily rhythms. Analyses of FAA conducted by chronobiologists indicates that it is controlled in part by a circadian clock, separate from the clock that controls activity rhythms synchronized by light dark cycles. When food is restricted to limited amount provided at a fixed time in the light period, circadian clock-controlled FAA emerges within a few days, while nocturnal activity typically decreases (depending on the type of activity being measured). The decrease in nocturnal activity does not appear to reflect changes in the phase or amplitude of the light-dark entrained circadian clock. For these reasons, it is preferable to refer to nocturnal activity as 'nocturnal activity', rather than as 'circadian-rhythm-related activity at night'. Chronobiological study of FAA is quite extensive, and the literature does include a number of methods papers, as well as studies that have employed video-based image analysis to quantify FAA.

Answer 4: Thank you for this comment on current findings regarding food anticipatory activity (FAA) in the context of circadian rhythms. Particularly relevant is the observation that the reduction in nocturnal activity during restricted feeding does not necessarily reflect a shift in the phase or amplitude of the light-entrained circadian clock. This interpretation is supported by multiple studies suggesting a functional dissociation between the suprachiasmatic nucleus (SCN), as the light-entrainable central oscillator, and a separate, food-entrainable oscillator (FEO) [2-4]. Notably, FAA persists even in SCN-lesioned animals [4, 5]. The observed decrease in nocturnal activity may reflect a redistribution of activity favoring FAA. Therefore, in future experiments, we plan to schedule the FAA period within the dark cycle to assess whether activity patterns correspondingly change in mice of the starvation-induced hyperactivity (SIH) model. In one of our previous studies, we demonstrated that chronic starvation led to circadian changes measured with the cosinor method associated with glial changes in the suprachiasmatic nucleus (SCN) [6]. We have specified the term circadian-rhythm-related activity to nocturnal activity as you recommended.

4 Discussion: "Analyses by chronobiologists indicate that FAA is controlled by a circadian clock distinct from the light-entrained clock regulating general activity rhythms [2, 3]. Therefore, circadian locomotor changes observed in starved mice may be driven by an increase in FAA. In a previous study, we showed that chronic starvation induces circadian alterations, assessed by cosinor analysis, which are associated with glial changes in the hypothalamus [6]. Accordingly, future studies will investigate whether these circadian changes depend on the timing of FAA." References 1. Klein, C., et al., Measuring Locomotor Activity and Behavioral Aspects of Rodents Living in the Home-Cage. Front Behav Neurosci, 2022. 16: p. 877323.

2. Mistlberger, R.E., Neurobiology of food anticipatory circadian rhythms. Physiol Behav, 2011. 104(4): p. 535-45.

3. Mistlberger, R.E., Food-anticipatory circadian rhythms: concepts and methods. Eur J Neurosci, 2009. 30(9): p. 1718-29.

4. Marchant, E.G. and R.E. Mistlberger, Anticipation and entrainment to feeding time in intact and SCN-ablated C57BL/6j mice. Brain Res, 1997. 765(2): p. 273-82.

5. Yoshihara, T., et al., Independence of feeding-associated circadian rhythm from light conditions and meal intervals in SCN lesioned rats. Neurosci Lett, 1997. 222(2): p. 95-8.

6. Zimmermann, A., et al., Changes in Circadian Rhythm in Chronically-Starved Mice Are Associated With Glial Cell Density Reduction in the Suprachiasmatic Nucleus. Int J Eat Disord, 2025.
